# Tonsillectomy postoperative analgesia study: protocol for a double-blinded, randomised controlled trial comparing ropivacaine and lidocaine as local anaesthesia regarding postoperative pain outcome after tonsillectomy

**DOI:** 10.1136/bmjopen-2026-123721

**Published:** 2026-11-09

**Authors:** Sebastian Ender, Thomas Machreich, Konstantin Emil Thiel, Gerd Rasp, Ildikó Takács

**Affiliations:** 1 Department of Otorhinolaryngology, Tauernklinikum, Zell am See, Salzburg, Austria; 2 PhD Medical Science Program, Paracelsus Medical University, Salzburg, Salzburg, Austria; 3 Department of Anesthesiology and Intensive Care, Tauernklinikum, Zell am See, Salzburg, Austria; 4 Core Facility Biostatistics, Paracelsus Medical University, Salzburg, Salzburg, Austria; 5 Department of Otorhinolaryngology, Head and Neck Surgery, County Hospital Salzburg University Hospital Salzburg, Salzburg, Salzburg, Austria

**Keywords:** Anaesthesia in otolaryngology, OTOLARYNGOLOGY, Head & neck surgery, PAIN MANAGEMENT, Randomized Controlled Trial

## Abstract

**Introduction:**

High pain intensity and prolonged duration of pain in the postoperative course after tonsillectomy is a significant challenge. The current standard local anaesthetic used perioperatively is lidocaine. However, recent studies suggest that the use of ropivacaine could significantly reduce postoperative pain. The Tonsillectomy Postoperative Analgesia Study (TOPAS) aims to improve postoperative pain management in the inpatient and outpatient phases.

**Methods and analysis:**

TOPAS is a prospective, randomised, double-blinded drug study with a continuous data collection beyond hospital discharge for a postoperative period of 14 days. It is designed to quantitatively evaluate pain intensity, analgesic requirements and the duration until the first rescue medication is taken across two groups over a 14-day observation period to improve postoperative pain management. Ropivacaine will be compared with lidocaine as a local anaesthetic being administered perioperatively while performing the tonsillectomy in patients suffering from recurrent or chronic tonsillitis. A total of 60 patients will be enrolled, split equally into the two groups. The primary endpoint is the mean postoperative pain intensity assessed via the visual analogue scale during the first 72 hours following tonsillectomy. The secondary endpoints are the mean postoperative pain intensity, assessed via visual analogue scale after discharge for an additional 11 days, mean analgesic consumption during the inpatient stay, mean analgesic consumption following hospital discharge for an additional 11 days and time to first rescue medication. Clinical follow-ups will be conducted 1 and 2 weeks postoperatively.

**Ethics and dissemination:**

This study has been approved by the Ethics Committee of Salzburg as well as the Austrian Federal Office for Safety in healthcare and is registered via the European Medicines Agency Clinical Trials Information System (EMA CTIS). Results will be submitted for publication and will be presented at scientific conferences.

**Trial registration number:**

EMA CTIS number 2025-5 20 820-44-00.

**STRENGTHS AND LIMITATIONS OF THIS STUDY**

- This is a prospective, randomised, double-blinded drug study with a continuous data collection beyond hospital discharge for a total period of 14 days.
- Standardised anaesthesia, surgical and postoperative protocols are used, with all procedures performed by a single operator to minimise potential confounding factors. Concurrently, findings may not be directly transferable to other techniques.
- During the outpatient phase, data quality relies on patient documentation, which partially limits the possibility of verification.
- Should high dropout rates occur in the outpatient setting, the study may yield non-significant results for its according secondary endpoints.

## Introduction

 Tonsillectomy is among the most common elective surgical procedures in otorhinolaryngology; however, postoperative pain remains a significant challenge despite modern surgical techniques. A primary issue in the postoperative course is the relatively high pain intensity, which frequently necessitates high analgesic consumption and can delay patient recovery. In addition to intensity, the duration of pain remains a significant challenge.^1–6^ Due to the sloughing of fibrin coatings as part of the natural healing process approximately 1 week postoperatively, patients experience a secondary peak in pain intensity during the outpatient recovery phase. This presents a particularly difficult situation for both the patient and the treating physician, as the continuous clinical monitoring, available in the inpatient setting is no longer present. These elevated pain levels, compared with other similar surgeries, are attributable to the specific anatomical site. Any contact of the surgical site with liquids or a food bolus inevitably triggers exacerbated pain. Furthermore, each act of swallowing involves movement and tissue stretching, which triggers additional increased pain. Consequently, there is ongoing clinical interest in optimising local anaesthetic techniques to improve pain management. This is of particular relevance as studies have already demonstrated a prolonged analgesic effect of ropivacaine extending beyond its documented pharmacological duration of action.^7 8^

The use of local anaesthetics for pain reduction is widespread, with lidocaine traditionally serving as the standard due to its rapid onset of action and established safety profile.^9 10^ However, its duration of action is limited, which can lead to a rapid and repeated need for analgesics in the early postoperative phase.^9^ Despite this limitation, lidocaine remains the standard of care due to its broad availability, cost-effectiveness and high degree of controllability.

Ropivacaine, a long-acting local anaesthetic characterised by an extended duration of action, a favourable sensory-to-motor blockade profile and low cardiotoxic potential, is increasingly being investigated as an alternative.^7 11–13^ Systematic reviews and meta-analyses suggest that infiltration with ropivacaine significantly reduces postoperative pain in both adults and children following tonsillectomy.^7 11^ Furthermore, randomised controlled trials indicate that ropivacaine provides a longer analgesic effect and a more favourable side-effect profile compared with lidocaine.^9 13^

Despite these positive indications, the clinical application of ropivacaine remains inconsistent. A recent meta-analysis on the use of local anaesthetics in paediatric tonsillectomy confirms the benefits of infiltration techniques but provides no definitive recommendation regarding the choice of a specific local anaesthetic; other studies similarly withhold a clear recommendation.^14–16^ Additionally, the procedure-specific postoperative pain management (PROSPECT) guidelines for postoperative pain management in tonsillectomy currently do not issue a recommendation for the use of ropivacaine.^17^ This gap in evidence-based recommendations underscores the necessity for further comparative studies between ropivacaine and the established standard, lidocaine.

To the best of our knowledge, there is currently no comparable study within the Central European region. Furthermore, to date, there is no equivalent study worldwide that evaluates pain outcomes after tonsillectomy up to 14 days postoperatively. As pain scores stay high after discharge, there is a critical need for studying the outpatient phase post surgery.

Therefore, the objective of this study is to systematically investigate the analgesic efficacy of ropivacaine compared with lidocaine in tonsillectomies, thereby strengthening the evidence base for the clinical use of ropivacaine in daily practice. Both local anaesthetics have been in standard clinical use for many years. Consequently, there are no concerns regarding ethical viability or the risk-benefit assessment.

This study is developed as an investigator-initiated study aimed at optimising the current pain management procedures for tonsillectomy. There are no external sponsors or commercial financial supporters for this project; therefore, our hospital, Tauernklinikum GmbH, is acting as the institutional sponsor of this trial. The sponsor holds no authority regarding the study design, conduct of the trial, analysis of the data and the subsequent publications.

## Methods and analysis

### Study design

The Tonsillectomy Postoperative Analgesia Study (TOPAS) is a double-blinded, randomised, single-centre comparative effectiveness trial aimed at evaluating the efficacy of ropivacaine compared with lidocaine used as local anaesthesia regarding postoperative pain in adults undergoing tonsillectomy, classified as a phase IV drug study. Adults with chronic tonsillitis are recruited from surrounding otorhinolaryngologists or directly from our otorhinolaryngology department. A total of 60 study participants will be randomised into two groups of 30 individuals each, with the lidocaine group defined as the control group and the ropivacaine group defined as the intervention group. Written informed consent will be obtained from the patient. Enrolled patients will undergo standard baseline assessment. All patients will undergo surgery done by one single surgeon, as well as having a standardised anaesthesia protocol. Pain scores will be documented four times a day within the in-clinic timeframe, as well as the period after discharge for a total of 14 days. The number of needed pain medication will be documented for the same period; furthermore, time till rescue medication is taken will be evaluated. Patients will be doing a check-up in our department or via phone call 1 week and 2 weeks postoperatively to maximise retention and collect interim data. Reporting of the TOPAS protocol adheres to the Standard Protocol Items for Randomised Trials (SPIRIT) statement as delineated in the online supplemental material SPIRIT Checklist.^18^

The trial started with recruitment after authorisation in June 2025 and is planned to conclude until end of Q1 2027.

### Study aims and endpoints

The objective of this study is to quantitatively evaluate pain intensity, analgesic requirements and the duration until the first rescue medication is taken across two groups over a 14-day observation period. Pain intensity will be documented four times a day using a visual analogue scale (VAS). Medication intake will be recorded as the number of analgesics administered, differentiating between two types of rescue medication. The timing of the first rescue medication will be defined as the duration from the end of surgery to administration, recorded in hours and minutes.

The primary endpoint is the mean postoperative pain intensity, assessed via VAS (0–10) during the first 72 hours following tonsillectomy. Measurements will be documented four times daily for a total of 12 assessments.

The secondary endpoints are the mean postoperative pain intensity (1) assessed via VAS (0–10) after discharge for an additional 11 days (postoperative days 4–14), with measurements documented four times daily; (2) mean analgesic consumption during the inpatient stay (days 1–4), differentiated by two distinct classes of analgesics; (3) mean analgesic consumption following hospital discharge (days 4–14), differentiated by two distinct classes of analgesics and (4) time to first rescue medication, measured in hours and minutes from the end of surgery to the time of administration.

### Patient protocols

To minimise potential bias arising from variation in surgical technique, clinical experience and operative speed, all procedures will be performed exclusively by the study author.

All patients will receive anaesthesia according to a predefined, standardised protocol described in box 1. Dosing of all anaesthetic agents is based on the ideal body weight of the respective patient. The anaesthetic management was standardised following consultations with internal anaesthesia staff and a review of current guidelines and relevant literature.^19–21^ This ensures full comparability between subjects and eliminates potential confounding factors associated with variations in anaesthetic technique or dosing.

Box 1 **Anesthesia protocol**

**Anesthesia**

- Sevoflurane anaesthesia with a minimum alveolar concentration of 1–1.3.
- Administration of fentanyl 3 µg/kg body weight (BW).
- Administration of diclofenac 1 mg/kg BW.
- Administration of dexamethasone 0.15 mg/kg BW.
- Administration of propofol 2–3 mg/kg BW.
- Administration of rocuronium 0.5 mg/kg BW.
- Administration of ephedrine 0.1–0.2 mg/kg BW if mean arterial pressure<65 mm Hg.
- Administration of elomel 10 ml/kg BW/h adjusted as needed.
- Administration of sugammadex 2 mg/kg BW in case of residual neuromuscular blockade at the end of surgery.
- Administration of ondansetron 0.1 mg/kg BW in patients with a known history of postoperative nausea and vomiting.
- No administration of any other analgesic or medication that influences the pain outcome.

**Recovery room**

- If necessary, administration of piritramide 0.05–0.1 mg/kg BW.

Postoperatively, pain levels will be monitored for a total of 14 days, covering both the inpatient stay and the period following discharge, using two customised pain protocols specifically developed for this study. On the day of surgery, data will be collected at 2, 4 and 6–8 hours post-intervention. Subsequently, assessments will be performed four times daily (morning, noon, evening and night). Pain intensity will be documented using the VAS (0–10). Additionally, the daily frequency of intravenous rescue medication use and any adverse events will be recorded in the study protocol. In the inpatient setting, documentation will be performed by trained nursing staff and during physician rounds.

Following discharge, patients will self-report their data using the same four-times-daily schedule. Patients will also record any instances of nocturnal awakening due to pain. Pain intensity (VAS 0–10), the frequency of the two per os rescue medications and any complications will continue to be documented in the outpatient study protocol. Patients will be educated and trained within the hospitalisation period.

Postoperative pain management will consist of a standardised fixed-dose regimen. In addition, a standardised rescue medication protocol will be established and documented accordingly.

### Study medication

#### Local anaesthesia

Lidocaine 0.5% with epinephrine (1:200 000) will be administered in the control group. It has been the standard local anaesthetic for tonsillectomies at our department for many years and is established as a global clinical standard.^9 14 15 22–27^

For the intervention group ropivacaine 0.5% with epinephrine (1:200 000) was selected to ensure adequate comparability through identical dosing concentrations. The concentration of 0.5% was favoured because it provides robust sensory anaesthesia while minimising the risk of motor blockade of the branches of the vagus and glossopharyngeal nerve, which is considered highly unlikely at this dosage.^28^

A pre-interventional application was selected to best prevent the activation of pain memory, as the local nerve block is established before the initial surgical pain stimulus occurs. Additionally, the added epinephrine induces vasoconstriction, which reduces intraoperative blood loss and enhances the visualisation of the surgical field.^26 27 29 30^

#### Analgesic therapy

Effective analgesia is critical for rapid convalescence, the minimisation of postoperative complications and the enhancement of patient satisfaction. Consequently, the pain management strategy is based on the following multimodal model:

Diclofenac: A well-established non-steroidal anti-inflammatory drug selected for its potent analgesic, antipyretic and anti-inflammatory properties.^31–35^ 50-mg film-coated tablets will be administered orally every 8 hours, three times daily within the hospital stay. Following discharge diclofenac 50-mg film-coated tablets will be taken orally on demand for pain levels of VAS≥3. It may be taken up to three times daily, maintaining a minimum interval of 6 hours between doses.

Benzydamine (Tantum Verde): An oral gargle solution that will be administered four times daily for the whole study duration of 14 days.

Metamizole: A strong non-opioid analgesic with additional spasmolytic effects, included as a standardised add-on therapy.^36 37^ 1 g will be administered intravenously as a rescue medication for pain levels of VAS≥5. It may be repeated up to four times daily, maintaining a minimum interval of 6 hours between doses within the hospital stay. Following discharge, metamizole medication will be changed to drops. 30 drops will be administered orally for persistent pain levels of VAS≥5. It may be taken up to four times daily, maintaining a minimum interval of 6 hours between doses.

Piritramide: An opioid used for the management of severe breakthrough pain which is characterised by its high degree of controllability and low emetogenic potential.^38 39^ 7.5 mg will be administered intravenously as a second line rescue medication for persisting pain levels of VAS≥7. It may be repeated up to three times daily, maintaining a minimum interval of 6 hours between doses within the hospital stay. In the event that the aforementioned opioid dose proves insufficient, the individual dose of piritramide may be doubled to 15 mg. In such cases, the administration will be recorded in the study protocol as a double dose and will be analysed statistically as two single doses. Piritramide administration is strictly limited to the inpatient setting.

The combination of diclofenac, benzydamine, metamizole and piritramide constitutes an effective, evidence-based multimodal regimen for pain management in patients aged 18 years and older following tonsillectomy.^17^ This strategy ensures that both mild and severe pain intensities are treated appropriately.

### Patient and public involvement

Prior postoperative feedback from patients undergoing tonsillectomy and well-known high and long lasting postoperative pain scores were the inspiration of the study and helped to shape the trial design. Specifically, patients who were involved in our preliminary testing, evaluating possible differences in postoperative pain using ropivacaine as a local anaesthetic, gave positive feedback with significantly longer lasting pain relief and significantly lower pain scores over the whole postoperative healing period. Therefore, making it essential for us to extend the evaluation period beyond the inpatient stay. This trial seeks to obtain more definitive data to determine if this method could improve clinical outcome measures.

### Registration

The trial is registered on euclinicaltrials.eu (2025-5 20 820-44-00).

### Recruitment

Otorhinolaryngologists are informed by mail with a self-made study factsheet about the trial. Therefore, admissions to our department can be done by them or by the patients themselves. Screening for eligibility will then be performed in our department.

### Screening and consent

Informed consent will be obtained prior to initiation of the study (refer to online supplemental material), as well as the standard informed consent for the operation and anaesthesia. At that personal visit in our department, screening for eligibility, matching all inclusion criteria and matching no exclusion criteria, will be performed.

### Inclusion criteria

The study will primarily include all patients aged 18 years and older with an otorhinolaryngological indication for bilateral tonsillectomy due to chronic tonsillitis.

### Exclusion criteria

Minor or pregnant patients, as well as patients who ingested analgesics within 24 hours prior to surgery or have a peritonsillar abscess will be excluded from the study. Moreover, patients with a known allergy or contraindication to the study medication, because of chronic illnesses or risk factors, must not be enrolled. If patients are administered drugs that cannot be taken in combination with the study medication, trial participation is not possible. For detailed exclusion criteria and prohibited concomitant medication, see boxes 2 and 3.

Box 2 **Exclusion criteria**

**General and clinical contraindications**

- Minor patients under the age of 18 or if there is a known pregnancy.
- Patients who have ingested analgesics within 24 hours prior to surgery.
- Prohibited concomitant medication that cannot be safely discontinued.
- Patients presenting with a peritonsillar abscess.
- Patients lacking the cognitive or linguistic capacity to understand the study procedures or to maintain the required documentation.

**Medical history and comorbidities**

- Known allergy or contraindication to any medication administered in the study.
- Severe cardiac, hepatic or renal insufficiency.
- High-grade cardiac arrhythmias or myocardial infarction.
- Hyperthyroidism.
- Diseases of the haematopoietic system.
- Peripheral arterial disease or cerebrovascular diseases.
- Active or recurrent gastrointestinal ulcers or bleeding.
- History of agranulocytosis.
- Hepatic porphyria.
- Glucose-6-phosphate dehydrogenase deficiency.
- Glaucoma.
- Analgesic-asthma syndrome.
- Chronic alcohol abuse.

**In-study exclusion**

- Patients requiring surgical revision due to postoperative haemorrhage.
- Present medication interaction that alters the efficacy or safety of the patient.
- Non-sufficient documentation or alteration of the medication used in the study.

Box 3 **Prohibited concomitant medication**

**Analgesia medication**

- Non-steroidal anti-inflammatory drugs.
- Non-opioid analgesics.
- Opioids.
- Local anaesthetics.

**Prohibited according to the summary of product characteristics**

- Tricyclic antidepressants.
- Monoamine oxidase inhibitors.
- Methotrexate.
- Ciclosporin.
- Tacrolimus.
- Other local anaesthetics.
- Class III antiarrhythmics structurally related to amide-type local anaesthetics.
- Bupropion.
- Efavirenz.
- Methadone.
- Valproate.
- Sertraline.
- Chlorpromazine.
- Central nervous system depressants.
- Serotonergic medicinal products.
- Cimetidine.

### Sample size

For the primary analysis, the mean VAS pain score during the first 72 hours (inpatient stay) will be evaluated. This mean value serves as the primary endpoint for a two-sample t-test designed to detect a hypothesised group difference of 2 points with a SD of 2.5 points, representing a large effect size, Cohen’s d=0.8. These assumptions are based on preliminary testing that was performed in our department, that showed a significant reduction in rescue analgesic demand over the 72 hours with an estimated mean VAS variance of 2.5, when ropivacaine was infiltrated perioperatively. Furthermore, comparable trials investigating local anaesthetic infiltration in tonsillectomy have demonstrated statistically and clinically significant differences in pain intensity using similar cohort sizes of around 30 per group.^9 13 25^

Based on these parameters, a sample size of 26 patients per group is required to achieve a significance level of α=0.05 and a statistical power of 80%.

To maintain sufficient statistical power in the event of an expected drop-out rate of up to 10%, a total sample size of 60 patients, 30 patients per group, is targeted.

Drop-out is defined as a cumulative amount of missing data exceeding 20% of the required assessments.

### Randomisation and blinding

The study will be conducted as a double-blind trial. To ensure concealment, the principal investigator (PI) will prepare 60 identical envelopes: 30 containing a slip labelled ‘Ropivacaine’ and 30 a slip labelled ‘Lidocaine’. These envelopes will be shuffled and kept in secure storage.

Preoperatively, the operating surgeon will draw one envelope per patient and hand it to the designated scrub nurse without opening it. The scrub nurse will then remove the slip and prepare the local anaesthetic in the absence of the surgeon. As the two local anaesthetics are optically indistinguishable, the surgeon remains blinded during administration.

For later identification and data linkage, the patient identification will be applied on the slip, which will then be returned to the envelope and resealed by the scrub nurse. After the operation, the envelope will be handed back to the PI, who will maintain the randomisation records. Once the final follow-up is complete, the data will be unblinded for documentation, solely by the PI or the operating surgeon. The PI is entirely independent of the surgical intervention and the surgeon has no involvement in the envelope preparation to remain the double-blinded status.

Patients will also be blinded throughout the entire follow-up period. Because the local anaesthetic is administered perioperatively under general anaesthesia, accidental unblinding is precluded.

### Treatment approach

This study will not affect the surgery or any of the medical care received before, during or after the surgery. All surgeries will be performed exclusively by the study author, an experienced surgeon. This approach is intended to eliminate inter-operator variability regarding surgical technique, clinical experience and operative speed, which might otherwise be introduced by the inclusion of multiple surgeons.

### Surgical procedure

The surgery is performed under general anaesthesia. Before mucosal incision is performed, a local anaesthetic is administered submucosally in the anterior and posterior tonsillar pillar. In total 10 mL are used, 5 mL per side. The strictly submucosal application gives direct visual confirmation of the proper injection site by localised mucosal blanching and tissue expansion, alongside missing blood aspiration prior to every injection. The procedure is performed as a cold-steel tonsillectomy, beginning on the right side. The technique involves a mucosal incision using a standard No. 15 scalpel, followed by blunt and sharp dissection using curved Metzenbaum dissecting scissors, angled up bipolar forceps and an Eve tonsil snare to stepwise deliver the tonsil. Following excision, the tonsillar fossa is packed with gauze swabs for temporary haemostasis, and the procedure is repeated on the left side in an identical manner. After the left tonsil is removed and the fossa is packed, definitive haemostasis is achieved using targeted electrocautery via the angled up bipolar forceps, starting on the right and proceeding to the left. The operation is concluded once complete haemostasis is confirmed.

### Follow-up visits and protocol monitoring

Clinical follow-ups are scheduled at one and 2 weeks postoperatively to monitor the documentation process. During these visits, solely the PI or the study author will review the entries, discuss any inconsistencies or observations with the patient and perform supplementary documentation if required. Any clinically relevant findings or abnormalities may be recorded in both the medical record and the study protocols. In addition to the protocol review, a physical clinical examination will be performed and recorded.

If a patient is unable or unwilling to attend the follow-up visits at the study centre, specifically due to a geographic constraint, the two assessments will be conducted via telemedicine. In such cases on discharge from the hospital, patients are provided with a pre-addressed and stamped envelope to facilitate the return of the finalised study protocol. A clinical follow-up examination with the referring otorhinolaryngologist 2 weeks postoperatively is mandatory for these patients. Results from that follow-up will be documented as part of the second telemedicinic assessment. As the results of the clinical follow-up are not part of the primary and secondary endpoints and adverse events will be documented via telemedicinic assessments, study results should not be distorted or influenced.

### Safety monitoring

To minimise the risk of local anaesthetic systemic toxicity (LAST), strict submucosal infiltration and direct visual control are performed. The total doses of 10 mL ropivacaine and lidocaine, both 50 mg, are substantially below the recommended maximum limits of 3–4 mg/kg for ropivacaine and 7 mg/kg for lidocaine. The co-administered epinephrine will function as an early indicator of accidental intravascular entry, as well as slowing the systemic absorption. Continuous intraoperative and postoperative monitoring is maintained. In the unlikely event of a LAST, standardised resuscitative measures according to the standardised LAST protocol will be initiated immediately.

All adverse events occurring during the study will be documented comprehensively and promptly. This documentation includes the nature of the event, onset, duration, severity, causality, actions taken and the final outcome. All relevant events will be reported by the PI to the sponsor of this trial, our hospital Tauernklinikum GmbH.

Serious adverse events (SAEs) that are not classified in the protocol as requiring immediate reporting will still be systematically recorded by the PI and submitted to the institutional sponsor as part of the safety evaluation. The following events are defined as SAEs not requiring immediate reporting: (1) postoperative haemorrhage during the inpatient stay or in the outpatient setting requiring readmission with or without surgical revision; (2) prolongation of the inpatient stay due to pain that cannot be managed by oral medication alone and (3) hospital readmission due to pain that cannot be managed by oral medication alone. These exceptions are justified as the aforementioned situations are recognised as common, general postoperative complications associated with tonsillectomy.

The sponsor is responsible for reporting suspected unexpected serious adverse reactions to the EudraVigilance database, the competent authorities and the involved Ethics Committee within the legally mandated timeframes.

In the event of an adverse reaction, targeted follow-up of the affected study participants will be conducted. The nature, duration and results of the follow-up will be documented to ensure a comprehensive safety assessment.

### Insurance and liability

A no-fault subject insurance has been concluded for this study in accordance with §40 of the Austrian Medicinal Products Act. This insurance guarantees that all participating subjects are covered for health impairments causally related to their participation in the study, regardless of whether the PI or the sponsor is at fault. The insurance coverage encompasses both direct and indirect health consequences that may result from the administration of the investigational medicinal product or from study-related procedures. Detailed insurance information is provided in the patient information and informed consent form, which is handed to each participant prior to study enrolment.

Furthermore, the PI maintains a valid professional liability insurance that covers occupational risks associated with conducting clinical trials. Additionally, a legal expenses insurance is in place to cover potential legal disputes related to the conduct of the study. All insurance policies comply with current national guidelines and regulatory requirements.

### Data collection and management

Parts of the study data will be recorded as routine pain medication documentation within the internal electronic health record system (Patidok with MedCaSol integration) in accordance with §§ 9 and 10 of the Austrian Hospitals and Spa Facilities Act (Krankenanstalten- und Kuranstaltengesetz - BGBI Nr. 1/1957 idF BGBI Nr.191/2003). Additional study-specific data collection will be performed using standardised protocols that are not integrated into the permanent medical record.

Collected data will be pseudonymised using the inpatient case number. While this allows for direct correlation if necessary, it ensures that data processing does not rely on sensitive personal identifiers. Digital data will be stored in an access-restricted document, accessible exclusively to the PI and the study author. Physical records will be kept in a secure, locked location, with access likewise restricted to the PI and the study author. Only these two individuals possess the means to re-identify the data if required.

All personnel involved in the study are bound by medical confidentiality and must comply with the General Data Protection Regulation (GDPR), as well as the relevant Austrian data protection adaptation laws in their currently valid versions.

There will be no transfer of data to external companies, and no data will be transmitted to countries outside of the European Union. For any subsequent publications, only pseudonymised or anonymised data will be used.

### Withdrawal

Study participants may withdraw their consent for the collection and processing of their data at any time without providing a reason. Following such a withdrawal, no further data will be collected.

In accordance with the GDPR, participants generally hold the rights to access, rectification, erasure, restriction of processing, data portability and objection. These rights apply insofar as they do not render the objectives of the study impossible or seriously impair them and provided that they do not conflict with other statutory regulations or legal requirements.

### Monitoring

Monitoring will be conducted using a risk-based approach. Responsibility for oversight lies with the Sponsor, represented by the Head of Quality Management at Tauernklinikum GmbH. To ensure the integrity of the oversight process, the monitor has no financial, scientific or other conflict of interest with the trial and is not directly involved in the conduct of the study, thereby maintaining strict operational independence.

The primary objectives of the monitoring activities are: (1) to protect the rights, safety and well-being of the study participants; (2) to ensure strict adherence to the study protocol and compliance with all regulatory requirements and (3) to guarantee the quality and integrity of the collected data.

All monitoring activities will be formally documented in written monitoring reports and archived accordingly. Any identified protocol deviations or relevant incidents will be evaluated, documented and, if necessary, reported to the competent authorities or the Ethics Committee.

### Data analysis

Sample size planning was conducted based on the core study parameters: expected effect sizes, significance level, statistical power, prior assumptions and the planned statistical models.

The primary endpoint is the postoperative pain score, assessed via VAS (0–10), recorded four times daily over a 14-day observation period.

The following statistical methods will be employed for data evaluation: Pain intensity (VAS): Analysis will be performed using generalised estimating equations (GEEs) with an ordinal logit link function to account for the longitudinal nature and ordinal scale of the pain scores. Analgesic consumption: The quantity of medication will be analysed using generalised linear mixed models assuming a negative binomial distribution, which is appropriate for overdispersed count data. Time to rescue medication: Data will be evaluated using Kaplan-Meier survival analysis and restricted mean survival time to quantify the duration until the first administration.

When necessary, a multiplicative adjustment of the significance level will be performed using the Holm-Bonferroni procedure. This ensures flexible yet conservative control of the error rate across all tested outcomes.

GEE models offer significant advantages in follow-up studies with missing data because they provide valid, unbiased estimates under the missing completely at random assumption without requiring you to specify the correct joint distribution of the repeated measures. Unlike traditional approaches that drop entire participants if a single time point is missing (listwise deletion), GEE uses all available data from every subject, maximising statistical power. Furthermore, when combined with weighted methods or multiple imputation, GEE can be easily extended to handle data that are missing at random, making it a highly robust and flexible choice for analysing longitudinal cohorts where attrition is common.

Statistical analysis will be conducted using R V.4.4.1 or later (R Core Team).

### Data sharing statement

Data sharing is not applicable to this article as the dataset has not been fully generated and has not been analysed during the current stage. However, upon study completion, de-identified individual participant data will be made available upon reasonable request to the corresponding author. A data sharing plan has been written (refer to online supplemental material). This plan, parts of the data, study protocol and trial information will be available via European Medicines Agency Clinical Trials Information System (EMA CTIS) (Trial ID: 2025-5 20 820-44-00) too.

## Ethics and dissemination

This clinical trial is conducted in strict accordance with the provisions of the study protocol, Regulation (EU) No 536/2014 (Clinical Trials Regulation) and the principles of Good Clinical Practice (GCP). Furthermore, the study is implemented in full compliance with the Declaration of Helsinki in its current applicable version, as well as all relevant national and international statutory regulations concerning protection of study participants.

All personnel involved in this research project are obligated to strictly adhere to the protocol and its appendices. Any protocol deviation must be thoroughly documented, justified and, if required, reported to the responsible Ethics Committee, the competent authority and the sponsor. Amendments to the protocol may only be implemented following prior approval by the competent authorities and Ethics Committees, unless immediate hazards to the participants necessitate urgent safety measures.

It is the responsibility of the PI to ensure that the study is conducted in accordance with GCP guidelines and the clinical trial protocol and to guarantee the accurate documentation of data. All data collected during the trial must be documented by authorised study staff or by the patients themselves. This requirement also applies to data from patients who are prematurely withdrawn or excluded from the study.

Given that the study uses two local anaesthetics that have been established as worldwide standards of care for many years, no health risks are anticipated beyond the standard inherent risks of the surgical procedure itself. Lidocaine and ropivacaine are routinely used for local infiltration to achieve a peripheral nerve block and thereby minimise pain perception. The local anaesthetics will be administered using the exact same technique as used during standardised infiltration for tonsillectomy. Consequently, there are no concerns regarding the ethical viability or the risk-benefit ratio of this intervention.

Aside from completing the study protocol, no additional burdens are expected for the study participants. It is possible that participation in this study will yield no direct, additional individual benefit to the patients.

This study has been approved by the Ethics Committee of Salzburg as well as the Austrian Federal Office for Safety in Health Care and is registered via the EMA CTIS (Registration Number: 2025-5 20 820-44-00). Written informed consent for study participation, as well as separate informed consent for the surgical procedure and anaesthesia, will be obtained from every patient prior to study enrolment.

We will submit trial results for publication to peer-reviewed journals and present results at one or more scientific conferences. No report or publication will contain any identifiable data that could lead to the participants’ identification.

## Supplementary material

10.1136/bmjopen-2026-123721 online supplemental file 1

10.1136/bmjopen-2026-123721 online supplemental file 2

10.1136/bmjopen-2026-123721 online supplemental file 3
