# Supplementary material for: Tonsillectomy postoperative analgesia study: protocol for a double-blinded, randomised controlled trial comparing ropivacaine and lidocaine as local anaesthesia regarding postoperative pain outcome after tonsillectomy
Source: BMJ Open. 2026 Nov 9;16(11):e123721. doi: 10.1136/bmjopen-2026-123721 (PMC13756231; doi:10.1136/bmjopen-2026-123721)
Supplement: Supplementary file 1 [file bmjopen-16-11-s002.pdf]

PatientInneninformation[1] und Einwilligungserklärung zur Teilnahme an der klinischen Prüfung

Vergleich zwischen der Verwendung von Ropivacain und Lidocain als Lokalanästhesie bezüglich des postoperativen Schmerzoutcomes nach Tonsillektomie

Sehr geehrte Prüfungsteilnehmerin, sehr geehrter Prüfungsteilnehmer!

Wir laden Sie ein an der oben genannten klinischen Prüfung teilzunehmen. Die Aufklärung darüber erfolgt in einem ausführlichen ärztlichen Gespräch.

Ihre Teilnahme an dieser klinischen Prüfung erfolgt freiwillig. Sie können jederzeit ohne Angabe von Gründen aus der Studie ausscheiden. Die Ablehnung der Teilnahme oder ein vorzeitiges Ausscheiden aus dieser Studie hat keine nachteiligen Folgen für Ihre medizinische Betreuung.

Klinische Prüfungen sind notwendig, um verlässliche neue medizinische Forschungsergebnisse zu gewinnen. Unverzichtbare Voraussetzung für die Durchführung einer klinischen Prüfung ist jedoch, dass Sie Ihr Einverständnis zur Teilnahme an dieser klinischen Prüfung schriftlich erklären. Bitte lesen Sie den folgenden Text als Ergänzung zum Informationsgespräch mit Ihrem Prüfarzt sorgfältig durch und zögern Sie nicht Fragen zu stellen.

Bitte unterschreiben Sie die Einwilligungserklärung nur

- wenn Sie Art und Ablauf der klinischen Prüfung vollständig verstanden haben,
- wenn Sie bereit sind, der Teilnahme zuzustimmen und
- wenn Sie sich über Ihre Rechte als Teilnehmer an dieser klinischen Prüfung im Klaren sind.

Zu dieser klinischen Prüfung, sowie zur Patienteninformation und Einwilligungserklärung wurde von der zuständigen Ethikkommission eine befürwortende Stellungnahme abgegeben.

1. Was ist der Zweck der klinischen Prüfung?

Der Zweck dieser klinischen Prüfung ist ist zu überprüfen, ob eine Verwendung eines langwirksamen im Vergleich zu einem kurzwirksamen lokalen Betäubungsmittels eine Verbesserung der Schmerzsituation nach der Operation mit sich bringt. Folgende Punkte werden vergleichen:

- Höhe der Schmerzen über 72h nach der Operation

[1] Wegen der besseren Lesbarkeit wird im weiteren Text zum Teil auf die gleichzeitige Verwendung weiblicher und männlicher Personenbegriffe verzichtet. Gemeint und angesprochen sind – sofern zutreffend – immer beide Geschlechter.

- Höhe der Schmerzen von Tag 4 bis Tag 14 nach der Operation
- Höhe der Schmerzmittelmenge über 72h nach der Operation
- Höhe der Schmerzmittelmenge von Tag 4 bis Tag 14 nach der Operation
- Dauer bis zur erstmalig notwendigen intravenösen Schmerzmittelgabe

2. Welche anderen Behandlungsmöglichkeiten gibt es?

Zur Behandlung stehen **stattdessen auch** die folgenden Möglichkeiten zur Verfügung: Standardmäßig erfolgt international die Verwendung des kurzwirksamen Lidocains während der Operation. Es gibt jedoch auch die Möglichkeit, die Operation ohne lokales Betäubungsmittel durchzuführen. Die Verwendung von weiteren anderen lokalen Betäubungsmitteln ist klinisch noch nicht gut erforscht. Eine Schmerztherapie nach der Operation mittels Tabletten und Infusionen erfolgt standardmäßig immer, auch im Rahmen der Studie.

3. Wie läuft die klinische Prüfung ab?

Diese klinische Prüfung wird an unserer Klinik durchgeführt, und es werden insgesamt ungefähr 60 Personen daran teilnehmen.

Vor Aufnahme in diese klinische Prüfung wird die Vorgeschichte Ihrer Krankheit erhoben, und Sie werden einer umfassenden ärztlichen Untersuchung unterzogen.

Ihre Teilnahme an dieser klinischen Prüfung wird voraussichtlich 2 Wochen dauern.

Im Rahmen dieser klinischen Prüfung wird Ropivacain (ein langwirksames lokales Betäubungsmittel) mit Lidocain (ein kurzwirksames lokales Betäubungsmittel), verglichen. Welche der Behandlungen Sie erhalten ist zufallsbedingt. Die Wahrscheinlichkeit eines der beiden Medikamente zu erhalten, beträgt jeweils 50%, steht also im Verhältnis 1:1.

Sie werden während der Operation entweder einmalig 10ml Ropivacain oder Lidocain, jeweils als Injektion zu 5ml pro Seite im Bereich um die Mandeln erhalten. Sie und Ihr Prüfarzt wissen nicht, welches Medikament Sie bekommen. Sollte es aber notwendig werden, kann Ihr Prüfarzt jederzeit in Erfahrung bringen, welches Medikament Sie erhalten haben.

Nach der Operation erfolgt die Schmerzmitteleinnahme wie folgt: Im Krankenhausaufenthalt bekommen Sie Schmerzmittel alle 8 Stunden (Diclofenac 50mg oral 3x tgl.). Reicht das nicht aus, um eine gute Schmerzsituation herzustellen (VAS ≥5 (Schmerzen von 0-10)), bekommen Sie ein weiteres Schmerzmittel bis zu 4x täglich (Novalgin 1g intravenös im Abstand von mindestens 6 Stunden). Bei weiterhin bestehenden starken Schmerzen (VAS ≥7) kann ein weiteres Medikament verabreicht werden (Dipidolor 7,5mg intravenös im Abstand von mindestens 6 Stunden). Zusätzlich erhalten Sie 4x tgl. eine betäubende Gugellösung. Nach der Entlassung erhalten Sie weiterhin Schmerzmittel, die Sie bei Schmerzen einnehmen sollen (Diclofenac 50mg oral bis zu 3x tgl., bei VAS ≥3, im Abstand von mindestens 6 Stunden), sowie weitere Schmerztropfen bei VAS ≥5 (Novalgin 30 Tropfen bis zu 4x tgl. im Abstand von mindestens 6 Stunden). Zusätzlich erhalten Sie 4x tgl. eine betäubende Gugellösung.

Eine Reihe von Untersuchungen und Eingriffen werden im Zuge Ihrer Behandlung durchgeführt, gleichgültig, ob Sie nun an dieser klinischen Prüfung teilnehmen oder nicht. Diese werden von Ihrem Prüfarzt im Rahmen des üblichen ärztlichen Aufklärungsgespräches mit Ihnen besprochen.

Folgende Maßnahmen werden ausschließlich aus Studiengründen durchgeführt:

Vor der Entlassung erhalten Sie ein Protokoll in dem standardisiert die aktuelle Schmerzsituation und eventuelle besondere Ereignisse dokumentiert werden. Über den genauen Ablauf werden Sie heute und erneut im Rahmen des stationären Aufenthalts informiert. Während dieser klinischen Prüfung werden im Abstand von 1 Woche die folgenden zwei Untersuchungen durchgeführt: klinische Kontrolle in der HNO-Ambulanz mit Besprechung der aktuellen Schmerzsituation und des (teil-)befüllten Protokolls eine, sowie zwei Wochen nach der Operation. Sie werden gebeten, hierzu jeweils in die HNO-Ambulanz des Tauernklinikums Zell am See zu kommen. Insgesamt sind 2 Besuche notwendig. Die Einhaltung der Besuchstermine, einschließlich der Anweisungen des Prüfarztes ist von entscheidender Bedeutung für den Erfolg dieser klinischen Prüfung.

4. Worum handelt es sich bei Lidocain, Ropivacain und Adrenalin?

Ropivacain ist ein Arzneimittel, welches zur lokalen Betäubung und einer somit verringerten Schmerzauslösung zugelassen ist.

Die Menge des einmalig verabreichten Ropivacains beträgt insgesamt maximal 10ml.

Lidocain ist ebenfalls ein Arzneimittel, welches zur lokalen Betäubung und einer somit verringerten Schmerzauslösung zugelassen ist.

Die Menge des einmalig verabreichten Lidocains beträgt ebenfalls insgesamt maximal 10ml.

Beiden Medikamenten ist Adrenalin in einer Dosierung von 1:200.000 beigemischt. Adrenalin ist ein Arzneimittel, das zur Beimengung von Lokalanästhetika zugelassen ist. Adrenalin wirkt verengend auf die Gefäße und hält somit das lokale Betäubungsmittel länger am Operationsort. Außerdem verringert es die Blutungsmenge während der Operation.

Beide lokalen Betäubungsmittel werden während der Operation vor der Mandelentfernung unter die Schleimhaut um die Mandeln gespritzt. So kann die Schmerzempfindung reduziert werden.

Alle drei Arzneimittel werden standardmäßig seit vielen Jahren in unterschiedlichen Fachbereichen eingesetzt.

5. Worin liegt der Nutzen einer Teilnahme an der Klinischen Prüfung?

Es ist möglich, dass Sie durch Ihre Teilnahme an dieser klinischen Studie keinen direkten zusätzlichen Nutzen für Ihre Gesundheit ziehen. Sie können mit Ihrer Teilnahme jedoch mithelfen, die Schmerztherapie für zukünftige Patienten wesentlich zu verbessern.

6. Gibt es Risiken, Beschwerden und Begleiterscheinungen?

Es können die im Rahmen dieser klinischen Studie durchgeführten Maßnahmen zu Beschwerden führen, oder sogar mit Risiken behaftet sein. Diese belaufen sich jedoch auf die allgemeinen Risiken der ohnehin geplanten Operation, wie Schmerzen, (Nach-) Blutung, Infektion, Schluckstörung, Verletzung umliegender Strukturen. Ein erhöhtes Risiko durch die Teilnahme an der Studie ist nicht zu erwarten. Mögliche Risiken/Nebenwirkungen der drei in der Studie verwendeten Medikamente sind allergische Reaktionen, Schwindel, Missempfindungen, veränderte Sinneseindrücke, Veränderung der Herzfrequenz und des Blutdrucks, Übelkeit, Erbrechen, Blutwertveränderungen, Herzrhythmusstörungen, Krämpfe.

7. Zusätzliche Einnahme von Arzneimitteln?

Es bestehen keine Vorschriften zur Einnahme von anderen Medikamenten, wie zum Beispiel Ihrer Dauermedikation. Lediglich bei einer Einnahme von Schmerzmittel wird bei Möglichkeit um eine Pausierung für 24h vor der Operation gebeten. Ist dies nicht möglich, kann keine Studienteilnahme erfolgen. Nach der Operation erfolgt die Schmerzmitteleinnahme wie folgt: Im Krankenhausaufenthalt bekommen Sie Schmerzmittel alle 8 Stunden (Diclofenac 50mg oral 3x tgl.). Reicht das nicht aus, um eine gute Schmerzsituation herzustellen (VAS ≥5 (Schmerzen von 0-10)), bekommen Sie ein weiteres Schmerzmittel bis zu 4x täglich (Novalgin 1g intravenös im Abstand von mindestens 6 Stunden). Bei weiterhin bestehenden starken Schmerzen (VAS ≥7) kann ein weiteres Medikament verabreicht werden (Dipidolor 7,5mg intravenös im Abstand von mindestens 6 Stunden). Zusätzlich erhalten Sie 4x tgl. eine betäubende Gugellösung. Nach der Entlassung erhalten Sie weiterhin Schmerzmittel, die Sie bei Schmerzen einnehmen sollen (Diclofenac 50mg oral bis zu 3x tgl., bei VAS ≥3, im Abstand von mindestens 6 Stunden), sowie weitere Schmerztropfen bei VAS ≥5 (Novalgin 30 Tropfen bis zu 4x tgl. im Abstand von mindestens 6 Stunden). Zusätzlich erhalten Sie 4x tgl. eine betäubende Gugellösung.

Die Einnahmen werden entsprechend protokolliert.

8. Hat die Teilnahme an der klinischen Prüfung sonstige Auswirkungen auf die Lebensführung und welche Verpflichtungen ergeben sich daraus?

Durch die Studie erfolgen zwei Nachkontrollen in der hiesigen HNO-Ambulanz. Diese Kontrollen werden ansonsten entweder im Krankenhaus, oder beim zuweisenden niedergelassenen HNO Facharzt durchgeführt. Im Rahmen der Studienteilnahme muss eine Dokumentation des aktuellen Schmerzes 4x täglich über 14 Tage durchgeführt werden. Besondere Ereignisse müssen dokumentiert werden. Ansonsten kommt es durch die Studienteilnahme zu keinen zusätzlichen Verpflichtungen, oder Einschränkungen. Auf die generellen Empfehlungen nach der Operation ist jedoch zu achten.

9. Was ist zu tun beim Auftreten von Symptomen, Begleiterscheinungen und/oder Verletzungen?

Sollten im Verlauf der klinischen Prüfung irgendwelche Symptome, Begleiterscheinungen oder Verletzungen auftreten, müssen Sie diese Ihrem Prüfarzt mitteilen, bei schwerwiegenden Begleiterscheinungen umgehend, ggf. telefonisch (Telefonnummern, etc. siehe unten).

10. Versicherung

Als Teilnehmer an dieser klinischen Prüfung besteht für Sie der gesetzlich vorgeschriebene verschuldensunabhängige Versicherungsschutz (Personenschadenversicherung gemäß § 40 Arzneimittelgesetz, der alle Schäden abdeckt, die an Ihrem Leben oder Ihrer Gesundheit durch die an Ihnen durchgeführten Maßnahmen der klinischen Prüfung verursacht werden können, mit Ausnahme von Schäden auf Grund von Veränderungen des Erbmaterials in Zellen der Keimbahn).

Die Versicherung wurde für Sie bei der Zürich Versicherungs-Aktiengesellschaft (kurz Zurich), Leopold-Ungar-Platz 2, 1190 Wien, Tel. 08000 80 80 80 unter der Polizzennummer WF-95131686-5 abgeschlossen. Auf Wunsch können Sie in die Versicherungsunterlagen Einsicht nehmen.

Im Schadensfall können Sie sich direkt an den Versicherer wenden und Ihre Ansprüche selbständig geltend machen. Für den Versicherungsvertrag ist österreichisches Recht anwendbar, die Versicherungsansprüche sind in Österreich einklagbar.

Zur Unterstützung können Sie sich auch an die Patientenanwaltschaft, Patientenvertretung, oder Patientenombudsschaft wenden.

Um den Versicherungsschutz nicht zu gefährden

- dürfen Sie sich während der Dauer der klinischen Prüfung einer anderen medizinischen Behandlung nur im Einvernehmen mit Ihrem behandelnden Prüfarzt unterziehen **(ausgenommen davon sind Notfälle).** Dies gilt auch für die zusätzliche Einnahme von Medikamenten oder die Teilnahme an einer anderen Studie.
- müssen Sie dem behandelnden Prüfarzt - oder der oben genannten Versicherungsgesellschaft - eine Gesundheitsschädigung, die als Folge der klinischen Prüfung eingetreten sein könnte, unverzüglich mitteilen.
- müssen Sie alles Zumutbare tun um Ursache, Hergang und Folgen des Versicherungsfalles aufzuklären und den entstandenen Schaden gering zu halten. Dazu gehört ggf. auch, dass Sie Ihre behandelnden Ärzte ermächtigen, vom Versicherer geforderte Auskünfte zu erteilen.

11. Informationen für gebärfähige Frauen – Schwangerschaftstest

Schwangere und stillende Frauen dürfen an dieser klinischen Prüfung NICHT teilnehmen.

12. Wann wird die klinische Prüfung vorzeitig beendet?

Sie können jederzeit auch ohne Angabe von Gründen, Ihre Teilnahmebereitschaft widerrufen und aus der klinischen Prüfung ausscheiden ohne dass Ihnen dadurch irgendwelche Nachteile für Ihre weitere medizinische Betreuung entstehen.

Ihr Prüfarzt wird Sie über alle neuen Erkenntnisse, die in Bezug auf diese klinische Prüfung bekannt werden, und für Sie wesentlich werden könnten, umgehend informieren. Auf dieser Basis können Sie dann Ihre Entscheidung zur **weiteren** Teilnahme an dieser klinischen Prüfung neu überdenken.

Es ist aber auch möglich, dass Ihr Prüfarzt entscheidet, Ihre Teilnahme an der klinischen Prüfung vorzeitig zu beenden, ohne vorher Ihr Einverständnis einzuholen. Die Gründe hierfür können sein:

a) Sie können den Erfordernissen der Klinischen Prüfung nicht entsprechen;

b) Ihr Prüfarzt hat den Eindruck, dass eine weitere Teilnahme an der klinischen Prüfung nicht in Ihrem Interesse ist;

Sofern Sie sich dazu entschließen, vorzeitig aus der klinischen Prüfung auszuscheiden, oder Ihre Teilnahme aus einem der oben genannten Gründe vorzeitig beendet wird, ist es für Ihre eigene Sicherheit wichtig, dass Sie sich einer normalen Kontrolluntersuchung unterziehen. Diese besteht meistens aus einer körperlichen klinischen Untersuchung.

13. Datenschutz

Im Rahmen dieser klinischen Prüfung werden Daten über Sie erhoben und verarbeitet. Es ist grundsätzlich zu unterscheiden zwischen

1) jenen personenbezogenen Daten, anhand derer eine Person direkt identifizierbar ist (z.B. Name, Geburtsdatum, Adresse, Sozialversicherungsnummer, Bildaufnahmen...),
2) pseudonymisierten personenbezogenen Daten, das sind Daten, bei denen alle Informationen, die direkte Rückschlüsse auf die konkrete Person zulassen, entweder entfernt, durch einen Code (z. B. eine Zahl) ersetzt oder (z.B. im Fall von Bildaufnahmen) unkenntlich gemacht werden. Es kann jedoch trotz Einhaltung dieser Maßnahmen nicht vollkommen ausgeschlossen werden, dass es unzulässigerweise zu einer Re-Identifizierung kommt.
3) anonymisierten Daten, bei denen eine Rückführung auf die konkrete Person ausgeschlossen werden kann.

Zugang zu den Daten, anhand derer Sie direkt identifizierbar sind (siehe Punkt 1), haben der Prüfarzt und andere Mitarbeiter des Prüfzentrums, die an der klinischen Prüfung oder Ihrer medizinischen Versorgung mitwirken. Zusätzlich können autorisierte und zur Verschwiegenheit verpflichtete Beauftragte des Sponsors dem Tauernklinikum Zell am See, sowie Beauftragte von in- und/ oder ausländischen Gesundheitsbehörden und jeweils zuständige Ethikkommissionen in diese Daten Einsicht nehmen, soweit dies für die Überprüfung der ordnungsgemäßen Durchführung der klinischen Prüfung notwendig ist. Sämtliche Personen, die Zugang zu diesen Daten erhalten, unterliegen im Umgang mit den Daten den jeweils geltenden nationalen Datenschutzbestimmungen und/oder der EU-Datenschutz-Grundverordnung (DSGVO).

Der Code, der eine Zuordnung der pseudonymisierten Daten zu Ihrer Person ermöglicht, wird nur an Ihrem Prüfzentrum aufbewahrt.

Eine Weitergabe der Daten erfolgt nur in pseudonymisierter Form.

Für etwaige Veröffentlichungen werden nur die pseudonymisierten Daten verwendet.

Im Rahmen dieser klinischen Prüfung ist keine Weitergabe von Daten in Länder außerhalb der EU (Drittland) vorgesehen.

Ihre Einwilligung bildet die Rechtsgrundlage für die Verarbeitung Ihrer personenbezogenen Daten. Sie können die Einwilligung zur Erhebung und Verarbeitung Ihrer Daten jederzeit ohne Begründung widerrufen. Nach Ihrem Widerruf werden keine weiteren Daten mehr über Sie erhoben. Die bis zum Widerruf erhobenen Daten können allerdings weiter im Rahmen dieser klinischen Prüfung verarbeitet werden. Wenn Sie mit der Verarbeitung Ihrer Daten nicht einverstanden sind, können Sie nicht an dieser klinischen Prüfung teilnehmen.

Nach der DSGVO stehen Ihnen grundsätzlich die Rechte auf Auskunft, Berichtigung, Löschung, Einschränkung der Verarbeitung, Datenübertragbarkeit und Widerspruch zu, soweit dies die Ziele der klinischen Prüfung nicht unmöglich macht oder ernsthaft beeinträchtigt und soweit dem nicht andere gesetzliche Vorschriften widersprechen.

Das gemäß DSGVO vorgesehene Recht auf Löschung Ihrer im Rahmen dieser klinischen Prüfung verarbeiteten Daten steht Ihnen aufgrund von Regelungen nach dem Arzneimittelgesetz nicht zu. Zusätzlich ist bei einer klinischen Prüfung nach dem Arzneimittelgesetz das Recht auf Datenübertragbarkeit außer Kraft gesetzt.

Die voraussichtliche Dauer der klinischen Prüfung ist in etwa ein Jahr. Die Dauer der Speicherung Ihrer Daten über das Ende oder den Abbruch der klinischen Prüfung hinaus ist durch Rechtsvorschriften geregelt (Arzneimittelstudie mindestens 25 Jahre).

Ihre erhobenen pseudonymisierten Daten können vom Studienzentrum/von der Prüfstelle bzw. Sponsor für zukünftige Forschungsprojekte verwendet werden. Die konkreten Fragestellungen können zum jetzigen Zeitpunkt jedoch noch nicht genannt werden. Ziel dieser Forschungsprojekte ist es, die Diagnostik zu verbessern, das Verständnis für die Entstehung von Krankheiten zu vertiefen, neue Behandlungsansätze zu finden und die medizinische Versorgung zu optimieren. Alle Forschungsprojekte, die am Menschen durchgeführt werden, müssen einer zuständigen Ethikkommission zur Begutachtung vorgelegt werden.

Falls Sie Fragen zum Umgang mit Ihren Daten in dieser klinischen Prüfung haben, wenden Sie sich zunächst an Ihren Prüfarzt. Dieser kann Ihr Anliegen ggf. an die Personen, die für den Datenschutz verantwortlich sind, weiterleiten.

Name, Adresse und Kontaktdaten des Sponsors:

Tauernklinikum Zell am See, Paracelsusstraße 8, 5700 Zell am See

Tel.: 050 2720

Datenschutzbeauftragter des Tauernklinikums Zell am See:

Mag. Siegfried Gruber: siegfried.gruber@opp-beratung.com

Sie haben das Recht, bei der österreichischen Datenschutzbehörde eine Beschwerde über den Umgang mit Ihren Daten einzubringen (www.dsb.gv.at; E-Mail: dsb@dsb.gv.at).

14. Entstehen für die Teilnehmer Kosten? Gibt es einen Kostenersatz oder eine Vergütung?

Durch Ihre Teilnahme an dieser klinischen Prüfung entstehen für Sie keine zusätzlichen Kosten.

15. Möglichkeit zur Diskussion weiterer Fragen

Für weitere Fragen im Zusammenhang mit dieser klinischen Prüfung stehen Ihnen Ihr Prüfarzt und seine Mitarbeiter gern zur Verfügung. Auch Fragen, die Ihre Rechte als Patient und Teilnehmer an dieser klinischen Prüfung betreffen, werden Ihnen gerne beantwortet.

Name der Kontaktperson: Dr. Sebastian Ender...

Ständig erreichbar unter: +43 676 6085567..

Name der Kontaktperson: Univ.Doz. Ildiko Takacs, PhD...

Ständig erreichbar unter: +43 664 5052188..

16. Wo kann ich weitere Informationen einholen?

Bei Fragen zur Einverständniserklärung, können Sie sich auch an die Salzburger Patientenvertretung wenden.

Salzburger Patientenvertretung

Sebastian-Stief-Gasse 2

5020 Salzburg

Tel.:	0662 8042-2030
Fax.:	0662 8042-3204
E-Mail:	patientenvertretung@salzburg.gv.at

Die EU-CT-Nummer, die diese klinische Prüfung innerhalb der EU eindeutig identifiziert, lautet:

2025-520820-44-00

https://euclinicaltrials.eu/

Es wird darauf hingewiesen, dass die Zusammenfassung der Ergebnisse der klinischen Prüfung und eine Zusammenfassung, die in einer für Laien verständlichen Sprache formuliert ist, unabhängig vom Ergebnis der klinischen Prüfung und wie in der Clinical Trials Regulation (EU) 536/2014 vorgesehen, in der EU-Datenbank eingesehen werden kann.

17. Sollten andere behandelnde Ärzte von der Teilnahme an der klinischen Prüfung informiert werden?

Andere behandelnde Ärzte müssen nicht über die Teilnahme informiert werden.

19. Einwilligungserklärung

Name des Prüfungsteilnehmers:

Geb.Datum:

Ich erkläre mich bereit, an der klinischen Prüfung "Vergleich zwischen der Verwendung von Ropivacain und Lidocain als Lokalanästhesie bezüglich des postoperativen Schmerzoutcomes nach Tonsillektomie"teilzunehmen. Ich bin darüber aufgeklärt worden, dass ich die Teilnahme ohne nachteilige Folgen, insbesondere für meine medizinische Betreuung, ablehnen kann.

Ich bin von Frau/Herrn (Dr.med.) ....ausführlich und verständlich über die klinische Prüfung, mögliche Belastungen und Risiken, sowie über Wesen, Bedeutung und Tragweite der klinischen Prüfung, die bestehende Versicherung sowie die sich für mich daraus ergebenden Anforderungen aufgeklärt worden. Ich habe darüber hinaus den Text dieser Patientenaufklärung und Einwilligungserklärung, die insgesamt 10 Seiten umfasst, gelesen. Aufgetretene Fragen wurden mir vom Prüfarzt verständlich und zufriedenstellend beantwortet. Ich hatte ausreichend Zeit, mich zu entscheiden. Ich habe zurzeit keine weiteren Fragen mehr.

Ich werde den ärztlichen Anordnungen, die für die Durchführung der klinischen Prüfung erforderlich sind, Folge leisten, behalte mir jedoch das Recht vor, meine freiwillige Mitwirkung jederzeit zu beenden, ohne dass mir daraus Nachteile, insbesondere für meine medizinische Betreuung, entstehen.

☐ Ich stimme der Teilnahme an dieser klinischen Prüfung ausdrücklich zu.

☐ Ich stimme ebenfalls zu, dass meine im Rahmen dieser klinischen Prüfung erhobenen Daten verarbeitet werden, wie in dieser Teilnehmerinformation und Einwilligungserklärung beschrieben.

Ich stimme weiters der Information meines Facharztes über meine Teilnahme an dieser klinischen Prüfung zu.

☐ ja ☐ nein

Eine Kopie dieser Teilnehmerinformation und Einwilligungserklärung habe ich erhalten. Das Original verbleibt beim Prüfarzt.

....
(Datum und Unterschrift des Patienten)

....
(Datum, Name und Unterschrift des verantwortlichen Prüfarztes)

(Der Prüfungsteilnehmer erhält eine unterschriebene Kopie der Patienteninformation und Einwilligungserklärung, das Original verbleibt im Studienordner des Prüfarztes.)
