# Supplementary material for: Tonsillectomy postoperative analgesia study: protocol for a double-blinded, randomised controlled trial comparing ropivacaine and lidocaine as local anaesthesia regarding postoperative pain outcome after tonsillectomy
Source: BMJ Open. 2026 Nov 9;16(11):e123721. doi: 10.1136/bmjopen-2026-123721 (PMC13756231; doi:10.1136/bmjopen-2026-123721)
Supplement: Supplementary file 2 [file bmjopen-2026-123721.reviewer_comments.pdf]

# PEER REVIEW HISTORY

BMJ Open publishes all reviews undertaken for accepted manuscripts. Reviewers are asked to complete a checklist review form and are provided with free text boxes to elaborate on their assessment. These free text comments are reproduced below.

## ARTICLE DETAILS

### Title (Provisional)

Tonsillectomy Postoperative Analgesia Study (TOPAS): protocol for a double-blinded, randomised controlled trial comparing Ropivacaine and Lidocaine as local anesthesia regarding postoperative pain outcome after Tonsillectomy

### Authors

Ender, Sebastian; Machreich, Thomas; Thiel, Konstantin Emil; Rasp, Gerd; Takács, Ildikó

---

## VERSION 1 - REVIEW

---

| | |
|---|---|
| **Reviewer** | **1** |
| **Name** | **Gan, Xiaoliang** |
| **Affiliation** | **Sun Yat-Sen University Zhongshan Ophthalmic Center State Key Laboratory of Ophthalmology** |
| **Date** | **02-Aug-2026** |
| **COI** | **None** |

---

Patients undergoing tonsillectomy often experience prolonged moderate to severe postoperative pain. Currently, the most commonly used method for pain management is local infiltration with lidocaine, supplemented by systemic analgesics.

Since lidocaine and ropivacaine have different mechanisms of action, previous studies have found conflicting differences between the two local anesthetics. This study aims to observe the differences in post-tonsillectomy pain relief between ropivacaine and lidocaine when used for local infiltration.

However, some details of the study design need to be improved or supplemented:

1. The study does not provide detailed information on the volume of local anesthetic injected or how the correct injection site is confirmed.

2. When patients experience moderate to severe pain, how will rescue analgesia be administered?

3. It is recommended to recalculate the sample size using time-weighted cumulative pain intensity. Additionally, the lack of a pilot study makes the sample size calculation inaccurate.

4. The maximum doses of the two local anesthetics differ, and the potential cardiotoxicity associated with each also differs. The manuscript needs to provide detailed explanations for the management of these side effects.

---


**Reviewer** **2**

**Name** **Košec, Andro**

**Affiliation** **University of Zagreb School of Medicine, Otorhinolaryngology and Head&Neck Surgery**

**Date** **22-Sep-2026**

**COI** **None**


---

The paper analyzes a postoperative analgesia regimen study protocol regarding tonsillectomy. A total of 60 participants will be enrolled and randomly assigned in a 1:1 ratio to receive either ropivacaine or lidocaine. The primary endpoint is the mean postoperative pain intensity measured on a Visual Analogue Scale (VAS 0–10) assessed four times daily across the first 72 hours postoperatively. Secondary endpoints comprise mean VAS pain scores evaluated four times daily from postoperative days 4 to 14, cumulative inpatient and outpatient analgesic consumption, and time to first rescue medication. Clinical follow-up assessments are scheduled at 1 week and 2 weeks postoperatively.

The statistical design appropriately handles repeated measurements and count data through Generalized Estimating Equations (GEE) with an ordinal logit link for longitudinal VAS scores, Generalized Linear Mixed Models (GLMM) with a negative binomial distribution for analgesic consumption counts, and Holm-Bonferroni corrections for multiple hypothesis testing. The protocol adheres to SPIRIT guidelines, and is prospectively registered (2025-520820-44-00).

However, there are several major methodological and scientific concerns:

Allocation sequence generation relies on the Principal Investigator preparing 60 paper envelopes (30 labeled Ropivacaine and 30 Lidocaine) and hand-shuffling them prior to secure storage. Hand-shuffling paper envelopes prepared directly by the PI presents a risk of sequence predictability and selection bias compared to computer-generated block randomization or centralized allocation.

The primary author/PI acts as the operating surgeon, prepares and stores the randomization envelopes, holds allocation records, and conducts follow-up protocol evaluations. Although the operating surgeon hands the sealed envelope to a scrub nurse to draw the medication in another room, having the PI handle the allocation sequence and outcome reviews creates potential blinding vulnerabilities.C

The power calculation assumes a group mean difference of 2.0 VAS points with a standard deviation of 2.5, requiring 26 patients per group for 80% power. If the true clinical effect size between local infiltration agents over 72 hours is moderate, the study will be substantially

underpowered. Provide preliminary data or literature justification for expecting a sustained 2-point reduction in mean VAS pain intensity across the initial 72 hours.

Requiring participants to log VAS scores 4 times daily for 14 days (56 separate entries per patient) imposes a heavy compliance burden. While cumulative missing data exceeding 20% is defined as a dropout, the protocol does not specify how missing entries under 20% will be handled in the GEE statistical model.

Relying on a single surgeon at one facility maximizes internal consistency but inherently limits the generalizability of the findings across varied surgical techniques, dissection devices (e.g., coblation vs. electrocautery), and clinical settings. Detail the specific dissection and hemostasis tools used during cold-steel tonsillectomy, and expand the limitations section to discuss external validity constraints.

I would recommend major revision aimed at these issues prior to re-review.

---

## VERSION 1 - AUTHOR RESPONSE

### Response to Reviewer 1

**Reviewer 1, Comment 1:**

*„The study does not provide detailed information on the volume of local anesthetic injected or how the correct injection site is confirmed.“*

**Author Response:**

Thank you for this important point. Detailed information was added to the manuscript regarding specification of volume as well as validation of the correct injection site to ensure reproducibility and safety. The „Surgical procedure“ section has been expanded.

**Changes in Manuscript:**

Page 15, lines 299–302 (Tracked-Change version):

„In total 10 ml are used, 5 ml per side. The strictly submucosal application gives direct visual confirmation of the proper injection site by localized mucosal blanching and tissue expansion, alongside missing blood aspiration prior to every injection.“

**Reviewer 1, Comment 2:**

*„When patients experience moderate to severe pain, how will rescue analgesia be administered?“*

**Author Response:**

Thank you for this valid question. Rescue medication will be administered intravenously within the hospital stay, and per os in the outpatient setting. This information was included in the original manuscript in the section „Analgesic therapy“ at line 200-220. However, we recognize that it may not have been sufficiently prominent. To ensure complete clarity for the reader, we have included it in the „Patient protocols“ section too.

**Changes in Manuscript:**

Page 7, lines 171 and 176 (Tracked-Change version):

„intravenous rescue medication" „per os rescue medication"

**Reviewer 1, Comment 3:**

*„It is recommended to recalculate the sample size using time-weighted cumulative pain intensity. Additionally, the lack of a pilot study makes the sample size calculation inaccurate."*

**Author Response:**

We thank both reviewers for these critical comments regarding our sample size calculation and the justification of our effect size. As these comments address the same core methodological aspect, we provide a unified response below.
We appreciate the opportunity to clarify the preliminary data and literature foundation behind our power estimation (target n = 30 per group accounting for dropouts):

1. Literature & MCID Justification:

A mean difference of 2 points on a 0–10 VAS scale represents the widely accepted Minimum Clinically Important Difference for acute postoperative pain management. Furthermore, comparable published randomized controlled trials investigating local anesthetic infiltration in tonsillectomy have successfully demonstrated statistically and clinically significant differences in pain intensity and cumulative analgesic consumption using similar cohort sizes of n = 30 per group.

- Ozkiriş M, Kapusuz Z, Saydam L. Comparison of ropivacaine, bupivacaine and lidocaine in the management of post-tonsillectomy pain. *Int J Pediatr Otorhinolaryngol*. Dec 2012;76(12):1831-4. doi:10.1016/j.ijporl.2012.09.011
- Nitin B, Gupta M. To Compare the Effects of Post-tonsillectomy Intra-operative Infiltration of Ropivacaine Versus Bupivacaine in Tonsillar Fossa. *Indian J Otolaryngol Head Neck Surg*. Apr 2024;76(2):1805-1818. doi:10.1007/s12070-023-04417-6
- Junaid M, Halim MS, Onali MAS, Qadeer S, Khan HU, Ali NS. Intraoperative Use of Analgesics in Tonsillar Fossa and Postoperative Evaluation with Visual analogue Scale Scores-A Prospective, Randomized, Placebo-Controlled, Double-Blind Clinical Trial. *Int Arch Otorhinolaryngol*. Jan 2020;24(1):e62-e67. doi:10.1055/s-0039-1684037

2. Preliminary Data:

Prior to finalizing this protocol, institutional preliminary evaluation revealed a notable reduction in total post-tonsillectomy rescue analgesic requirements between the local anesthetic regimens. While analgesic consumption cannot be directly converted 1:1 to VAS scores, it served as an objective surrogate indicator of pain control. Furthermore, initial preliminary observations indicated a group mean difference in pain scores of around 2 VAS points with a standard deviation of approximately 2.5, directly informing our standard deviation assumptions.

3. Statistical Clarification:

Our primary outcome analysis utilizes a Generalized Estimating Equations (GEE) framework (and a mixed-effects model as sensitivity analysis) across the repeated longitudinal time points over 72 hours. Evaluating the treatment effect longitudinally across multiple time points per patient provides substantially higher statistical power than a single cross-sectional comparison, making n = 30 per group sufficient to detect a moderate-to-large effect size without being underpowered.

We have updated the „Sample size“ section in the manuscript to explicitly cite the supporting literature and provide the rationale for the expected effect size.

**Changes in Manuscript:**

Page 14, lines 262–268 (Tracked-Change version):

„These assumptions are based on preliminary testing, that was performed in our department, that showed a significant reduction in rescue analgesic demand over the 72 hours with an estimated mean VAS variance of 2.5, when ropivacaine was infiltrated perioperatively. Furthermore, comparable trials investigating local anesthetic infiltration in tonsillectomy have demonstrated statistically and clinically significant differences in pain intensitiy using similar cohort sizes of around 30 per group.[9,13,25]“

**Reviewer 1, Comment 4:**

*„The maximum doses of the two local anesthetics differ, and the potential cardiotoxicity associated with each also differs. The manuscript needs to provide detailed explanations for the management of these side effects.“*

**Author Response:**

Thank you for highlighting this important safety aspect. We fully agree that local anaesthetic systemic toxicity and potential cardiotoxicity require clear risk assessment and predefined emergency protocols. To address this, we would like to clarify the administered doses in relation to established toxicity thresholds:

1. Dose Safety Margin: The protocol mandates a fixed, volume-matched infiltration of 10 ml peritonsillarly (5 ml per side) under direct visual control. This corresponds to:
    - Ropivacaine 0.5% (50 mg total) with Adrenaline 1:200.000: Well below the recommended maximum single dose of 225–300 mg or 3–4 mg/kg.
    - Lidocaine 0.5% (50 mg total) with Adrenaline 1:200.000: Well below the maximum dose of 500 mg or 7 mg/kg.
2. Administration Technique: Strict submucosal, peritonsillar infiltration under direct visualization minimizes the risk of accidental intravascular injection. Furthermore, the addition of adrenaline acts as an intravascular marker and reduces systemic absorption.

To ensure maximum patient safety and satisfy editorial expectations, we have expanded the „Safety monitoring“ section to explicitly detail the monitoring procedures and the management protocol for local anaesthetic systemic toxicity.

**Changes in Manuscript:**

Page 16-17, lines 327–333 (Tracked-Change version):

„To minimize the risk of local anaesthetic systemic toxicity (LAST), strict submucosal infiltration und direct visual control is performed. The total doses of 10 ml Ropivacaine and Lidocaine, both 50 mg, are substantially below the recommended maximum limits of 3-4 mg/kg for Ropivacaine and 7 mg/kg for Lidocaine. The co-administered Epinephrine will function as an early indicator of accidental intravascular entry, as well as slowing the systemic absorption. Continuous intraoperative and postoperative monitoring is maintained. In the unlikely event of a LAST, standardized resuscitative measures according to the standardised LAST protocol will be initiated immediately.“

## Response to Reviewer 2

**Reviewer 2, Comment 1:**

*„Allocation sequence generation relies on the Principal Investigator preparing 60 paper envelopes (30 labeled Ropivacaine and 30 Lidocaine) and hand-shuffling them prior to secure storage. Hand-shuffling paper envelopes prepared directly by the PI presents a risk of sequence predictability and selection bias compared to computer-generated block randomization or centralized allocation.*

*The primary author/PI acts as the operating surgeon, prepares and stores the randomization envelopes, holds allocation records, and conducts follow-up protocol evaluations. Although the operating surgeon hands the sealed envelope to a scrub nurse to draw the medication in another room, having the PI handle the allocation sequence and outcome reviews creates potential blinding vulnerabilities."*

**Author Response:**

We fully agree with the reviewer that a web-based computer randomisation system represents the gold standard for clinical studies. However, as the trial has already started and is currently active under the approved trial protocol, converting the allocation methodology now is methodologically unfeasible and will introduce protocol heterogenerity.

Nevertheless, we would like to clarify a crucial operational detail regarding the separate roles in our study protocol:

1. Strict Separation of Roles (PI vs. Operating Surgeon):

The PI, who generated the sequence and prepared the opaque, sealed envelopes prior to study initiation, is completely independent of the surgical team and does not perform the surgical procedures. The operating surgeon was not involved in the preparation of the envelopes and has no knowledge of the allocation sequence.

2. Complete Blinding & Allocation Safeguards:

- The pre-prepared, identical, opaque envelopes contain no external identifying markings.
- Prior to surgery, the operating surgeon selects an envelope from the secure storage. Because the surgeon was not involved in sequence generation or envelope preparation, total allocation concealment is maintained.
- The envelope is handed directly to an independent scrub nurse, who opens it and draws up the study medication in a separate preparation room.
- Neither the operating surgeon, the outcome assessors, nor the patient are aware of the group assignment at any point during surgery, recovery, or follow-up.

We have updated the „Randomisation and blinding" section to explicitly describe this clear division of responsibilities.

**Changes in Manuscript:**

Page 15, lines 287–288 (Tracked-Change version):

„The PI is entirely independent of the surgical intervention and the surgeon has no involvement in the envelope preparation to remain the double-blinded status."

**Reviewer 2, Comment 2:**

*„The power calculation assumes a group mean difference of 2.0 VAS points with a standard deviation of 2.5, requiring 26 patients per group for 80% power. If the true clinical effect size between local infiltration agents over 72 hours is moderate, the study will be substantially underpowered. Provide preliminary data or literature justification for expecting a sustained 2-point reduction in mean VAS pain intensity across the initial 72 hours.“*

**Author Response:**

We thank the reviewer for this comment. Please refer to our detailed response to Reviewer 1 (Comment 3), where we address the justification for our sample size calculation, citing preliminary institutional data, established MCID values, and comparable literature in detail.

**Reviewer 2, Comment 3:**

*„Requiring participants to log VAS scores 4 times daily for 14 days (56 separate entries per patient) imposes a heavy compliance burden. While cumulative missing data exceeding 20% is defined as a dropout, the protocol does not specify how missing entries under 20% will be handled in the GEE statistical model.“*

**Author Response:**

We thank the reviewer for this important comment regarding patient compliance and possible resulting statistical challanges in longitudinal data analysis. To clarify the handeling of missing data in the GEE statistical model, the „Data analysis“ section of the manuscript has been revised.

**Changes in Manuscript:**

Page 20, lines 417–424 (Tracked-Change version):

„Generalized Estimating Equations (GEE) models offer significant advantages in follow-up (FU) studies with missing data because they provide valid, unbiased estimates under the Missing Completely at Random (MCAR) assumption without requiring you to specify the correct joint distribution of the repeated measures. Unlike traditional approaches that drop entire participants if a single time point is missing (listwise deletion), GEE utilizes all available data from every subject, maximizing statistical power. Furthermore, when combined with weighted methods (WGEE) or multiple imputation, GEE can be easily extended to handle data that are Missing at Random (MAR), making it a highly robust and flexible choice for analyzing longitudinal cohorts where attrition is common.“

**Reviewer 2, Comment 4:**

*„Relying on a single surgeon at one facility maximizes internal consistency but inherently limits the generalizability of the findings across varied surgical techniques, dissection devices (e.g., coblation vs. electrocautery), and clinical settings. Detail the specific dissection and hemostasis tools used during cold-steel tonsillectomy, and expand the limitations section to discuss external validity constraints“*

**Author Response:**

We agree with the reviewer’s assessment of this methodological trade-off. Performing all procedures by a single surgeon using a standardized technique at a single centre was deliberately chosen to

maximize internal validity by eliminating inter-operator variability, differences in tissue handling, and surgical experience as potential confounding factors.

However, we fully acknowledge that this approach inherently constrains the external validity and generalizability of our findings across alternative surgical modalities (e.g., coblation, cold steel dissection) and broader clinical environments.

To ensure transparency, we have expanded that Limitation to the „Strengths and limitations of this study" section to explicitly address this matter.

**Changes in Manuscript:**

Page 3, lines 65–66 (Tracked-Change version):

„Standardized anesthesia, surgical, and postoperative protocols are used, with all procedures performed by a single operator to minimize potential confounding factors. Concurrently, findings may not be directly transferable to other techniques."

**Reviewer 2, Comment 5:**

*„Detail the specific dissection and hemostasis tools used during cold-steel tonsillectomy, and expand the limitations section to discuss external validity constraints."*

**Author Response:**

Thank you for this important point. As already mentioned in Comment 4, external validity constraints have been adressed. We have further refined the description of the surgical tools in the „Surgical procedure" section to specify the exact cold-steel and hemostatic instrumentation used.

**Changes in Manuscript:**

Page 3 and 15-16, lines 65–67 and 303–309 (Tracked-Change version):

„Standardized anesthesia, surgical, and postoperative protocols are used, with all procedures performed by a single operator to minimize potential confounding factors. Concurrently, findings may not be directly transferable to other techniques."

„The technique involves a mucosal incision using a standard No. 15 scalpel, followed by blunt and sharp dissection using curved Metzenbaum dissecting scissors, angled up bipolar forceps, and an Eve tonsil snare to step-wise deliver the tonsil. Following excision, the tonsillar fossa is packed with gauze swabs for temporary hemostasis, and the procedure is repeated on the left side in an identical manner. After the left tonsil is removed and the fossa is packed, definitive hemostasis is achieved using trageted electrocautery via the angled up bipolar forceps, starting on the right and proceeding to the left."

---

## VERSION 2 - REVIEW

---

**Reviewer** 2

**Name** Košec, Andro

**Affiliation** University of Zagreb School of Medicine, Otorhinolaryngology and Head&Neck Surgery

**Date** 14-Oct-2026

## COI

The authors have undertaken a comprehensive revision and I would now support the manuscript for publication.
