# Supplementary material for: Tonsillectomy postoperative analgesia study: protocol for a double-blinded, randomised controlled trial comparing ropivacaine and lidocaine as local anaesthesia regarding postoperative pain outcome after tonsillectomy
Source: BMJ Open. 2026 Nov 9;16(11):e123721. doi: 10.1136/bmjopen-2026-123721 (PMC13756231; doi:10.1136/bmjopen-2026-123721)
Supplement: Supplementary file 3 [file bmjopen-16-11-s001.docx]

**Data Sharing Plan**

**Trial Title: Comparison between Ropivacain and Lidocain for post-tonsillectomy pain**

**Trial Registration ID: 2025-520820-44-00 via EMA CTIS**

**Corresponding Author: Sebastian Ender; Department of Otorhinolaryngology, Tauernklinikum Zell am See, Zell am See, Austria;** [**sebastian.ender@stud.pmu.ac.at**](mailto:sebastian.ender@stud.pmu.ac.at)

**1. Statement on IPD Sharing**

De-identified individual participant data underlying the primary and secondary outcomes of this trial will be made available to qualified researchers upon reasonable request.
Furthermore trial data, study protocol and trial informations will be available via EMA CTIS (Trial ID: 2025-520820-44-00).

**2. Available Documents and Data**

- De-identified participant dataset
- Data dictionary
- Full study protocol
- Statistical Analysis Plan
- Statistical analysis code

**3. Timeframe of Availability**

Data will become available starting 3 months following the publication of the primary trial results in a peer-reviewed journal and will remain accessible for 5 years.

**4. Access Criteria and Restrictions**

- Location Constraint: Access is strictly limited to researchers based within member states of the European Union or European Economic Area to maintain compliance with GDPR.
- Purpose: Data will be shared exclusively for methodologically sound research proposals

**5. Access Mechanism and Data Use Agreement**

Interested researchers should submit a formal request and research proposal to the corresponding author ([sebastian.ender@stud.pmu.ac.at](mailto:sebastian.ender@stud.pmu.ac.at)). Approval requires the execution of a formal Data Use Agreement governing data security, confidential processing, and strict adherence to GDPR regulations.
